# Antiamnesic Effects of Feralolide Isolated from *Aloe vera* Resin Miller against Learning Impairments Induced in Mice

**DOI:** 10.3390/antiox12010161

**Published:** 2023-01-10

**Authors:** Imran Khan, Tapan Kumar Mohanta, Nuzhat Ihsan, Sobia Ahsan Halim, Ajmal Khan, Najeeb Ur Rehman, Faizullah Khan, Asaad Khalid, Ashraf N. Abdalla, Nasiara Karim, Ahmed Al-Harrasi

**Affiliations:** 1 Department of Pharmacy, University of Swabi KP, Swabi 94640, Pakistan; 2 Natural & Medical Sciences Research Center, University of Nizwa, Birkat Al Mauz, P.O. Box 33, Nizwa 616, Oman; 3 Department of Pharmacy, Abdul Wali Khan University Mardan, Mardan 23200, Pakistan; 4 Substance Abuse and Toxicology Research Center, Jazan University, P.O. Box 114, Jazan 45142, Saudi Arabia; 5 Medicinal and Aromatic Plants and Traditional Medicine Research Institute, National Center for Research, Khartoum P.O. Box 2404, Sudan; 6 Department of Pharmacology and Toxicology, College of Pharmacy, Umm Al-Qura University, P.O. Box 13578, Makkah 21955, Saudi Arabia; 7 Department of Pharmacy, University of Peshawar, K.P., Peshawar 25120, Pakistan

**Keywords:** feralolide, antiamnesic agent, scopolamine-induced mice, elevated plus maze test, ABTS, DPPH

## Abstract

Feralolide, a dihydroisocoumarin, was isolated from the methanolic extract of resin of *Aloe vera*. The present study aims to investigate the in vivo ability of feralolide to ameliorate memory impairment induced by scopolamine using a battery of in vitro assays, such as antioxidant and acetylcholinesterase (AChE) and butyrylcholinesterase (BuChE) inhibition, and in vivo animal models, including elevated plus maze, Morris water maze, passive avoidance, and novel object recognition tests. Feralolide caused a concentration-dependent inhibition of AChE and BuChE enzymes with IC_50_ values of 55 and 52 μg/mL, respectively, and antioxidant activity against 2,2-diphenyl-1-picrylhydrazyl (DPPH) and 2, 2′-azinobis-3-ethylbenzothiazoline-6-sulfonic acid (ABTS) with IC_50_ values 170 and 220 μg/mL, respectively. Feralolide reversed the scopolamine-induced amnesia as indicated by a dose-dependent decrease in escape latency, path length, and passing frequency in the Morris water maze test compared with the relevant control. The compound also significantly increased the discrimination index in a dose-dependent manner in NORT and decreased transfer latency in EPM, reflective of its memory-enhancing effect. Furthermore, feralolide also caused significant dose-dependent elevation in the step-down latency (SDL) in the passive avoidance test. The results indicated that feralolide might be a helpful memory restorative mediator in treating cognitive disorders such as Alzheimer’s disease.

## 1. Introduction

Dementia is a general term used to describe a group of symptoms affecting memory, thinking, and social and intellectual abilities severely enough to interfere with your daily life.

Dementia also includes other common types, such as Alzheimer’s disease (AD), dementia with Lewy bodies, cerebrovascular dementia, Parkinson’s disease, and frontotemporal dementia [1,2]. The most common type is AD, which is responsible for 50–60% of all cases). AD is a neurodegenerative disease with slow onset and progressive impairment of cognitive and behavioral functions, including memory attention, comprehension, reasoning, language, and judgment [3]. The initial symptom is usually memory loss. In AD, the most acceptable therapeutic approach has been the usage of acetylcholinesterase inhibitors that normally block the activity of acetylcholinesterase breakdown acetylcholine (ACh). This enzyme inhibition prevents the breakdown of the ACh that is known to be deficient in the AD brain. Although these drugs will be effective in the provision of symptomatic treatment, these drugs are non-selective. They may lead to the overstimulation of cholinergic systems throughout the body and cause a variety of cholinergic manifestations [4]. The currently available acetylcholine esterase AChEIs have uncertain clinical significance and are associated with serious adverse effects [5]. Thus, these therapeutic limitations in managing Alzheimer’s disease necessitate discovering and developing new and better drugs for this devastating disorder.

*Aloe vera* (L.) Burm. F. (Liliaceae) belongs to the genus *Aloe* (600 species) and is used to treat various ailments, including asthma, arthritis, ulcers, Crohn’s disease, skin irritations, antiseptic, hypoglycemic, germicidal, anti-inflammatory, blood purifier, ulcerative colitis, burns, and wound healing as well as in many formulations of health, medical, and cosmetic purposes. Most of the anthraquinones (aloin, emodin, and aloe-emodin), traditionally known as laxatives, isolated from aloe species, act as analgesics, antibacterials, and antivirals [6]. Previous findings indicated that aloe-emodin, the most active component of aloe species, could have neuroprotective effects against Alzheimer’s disease (AD) via inhibiting the activity of AChE and modulating oxidative stress [7]. A recent study on feralolide showed potent urease [8], weak α-glucosidase enzyme inhibition, and antioxidant effects. The compound attributed concentration-dependent antiproliferative effects on breast cancer cells (MDA-MB-231) and ovarian cancer cells (SKOV-3) [9]

Since there is no report, to the best of our knowledge, available on the scopolamine-induced memory impairment in mice concerning feralolide, which prompted the investigation of the isolated compound [10], the present study aimed to evaluate the possible memory-enhancing properties of the compound using various in vitro assays, in vivo, and in silico assays.

## 2. Materials and Methods

### 2.1. Chemicals

Acetylcholine iodide (Sigma-Aldrich, Gillingham, UK), butyrylcholine iodide (Sigma-Aldrich, Schaffhausen, Switzerland), and 5,5-dithio-bis-nitrobenzoic acid (DTNB) (Sigma-Aldrich, Taufkirchen, Germany) were purchased from local suppliers. Electric eel acetylcholinesterase (type-VI-S) and equine butyrylcholinesterase (Sigma-Aldrich, St. Louis, MO, USA), DPPH (2,2-diphenyl-1-picrylhydrazyl) from (Sigma-Aldrich, USA) and ABTS (2, 2′-azinobis-3-ethylbenzothiazoline-6-sulfonic acid) were purchased from Sigma-Aldrich, Germany and HPLC grade methanol was purchased from (Sigma-Aldrich, Gillingham, UK). Donepezil and scopolamine were purchased from Sigma (St. Louis, MO, USA). All chemicals and solvents used were of analytical grades.

### 2.2. Animals

A selected number of healthy albino mice weighing up to 20–30 g were used in the study. The animals were housed in several groups in individual cages made from stainless steel with softwood shavings as bedding and were provided with water and a standard pellet diet. They were maintained under normal laboratory conditions and at 12 h light-dark cycle. Activities were carried out according to the accepted guidelines of the Animal (Scientific Procedures) Act UK 1986 [11].

### 2.3. Sample Collection and Identification

The resin of *A. vera* was purchased from a local market (Souq) in Nizwa, Oman. The sample was identified by Mr. Saif Al-Hatmi (plant taxonomist) at the Oman Botanical Garden, Muscat. A voucher specimen (AFS-08/2016) was deposited in the herbarium of the Natural and Medical Sciences Research Centre, University of Nizwa, Oman.

### 2.4. Extraction and Isolation

**Figure 1:**
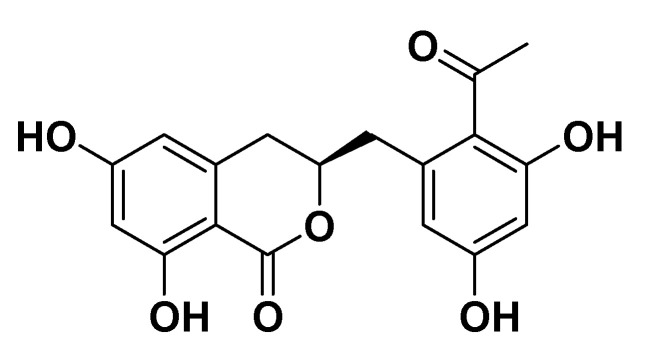
Structure of feralolide.

The air-dried and finely powdered resin (1042 g) of *A. vera* was extracted with methanol (2.5 L) at room temperature (3 × 15 days). Evaporation of the methanol under reduced pressure at 45 °C yielded a crude MeOH extract (987.0 g), which after being suspended in water was successively fractionated into *n*-hexane (0.9 g), dichloromethane (8.5 g), ethyl acetate (73.2 g), *n*-butanol (602.0 g), and aqueous fractions (289.0 g) based on an increasing polarity of organic solvents. Ethyl acetate fraction was subjected to a silica gel chromatographic column (70–230 mesh; Merck, Darmstadt, Germany) and sequentially eluted using *n*-hexane-EtOAc, EtOAc-MeOH, and pure MeOH with 10% increments of the polarity of the eluent to afford 14 fractions (Fr. 1–14). Fraction 16 (Fr. 5–8), eluted with the 40–60% EtOAc-*n*-hexane eluent, was further subjected to repeated column chromatography to afford feralolide (150 mg) [9] (Figure 1).

### 2.5. Assessment of Feralolide in In Vitro Assays

#### 2.5.1. DPPH Radical Scavenging Assay

The compound’s total free radical scavenging capacity was carried out according to the previously reported method [12] with a slight modification by using stable DPPH (2,2diphenyl-1-picrylhydrazyl radical), which has a maximum of 515 nm of absorption. By dissolving 2.4 mg of DPPH, a solution of the radical was prepared in 100 mL of methanol. A test solution of (5 μL) was added to the 3.995 mL of a methanolic DPPH. The mixture was vigorously shaken and then kept at room temperature for about 30 min in the dark. Absorbance at 515 nm of the reaction mixture was measured spectrophotometrically. Measurement of the absorbance of DPPH radical, without antioxidant, i.e., blank, was also performed. All the determinations were performed in triplicate form. The scavenging capability of the DPPH radical was also calculated by using a specific equation [13].

The formula for scavenging free radicals by a compound:

$$\%\;Radical\;scavenging=\frac{A-B}{A}\times 100$$

A = control absorbance; B = sample absorbance.

Control value: A = 0.723, Abs. on UV–visible spectrophotometer.

#### 2.5.2. ABTS Radical Scavenging Assay

Free radical scavenging, the compound’s activity was determined by 2,2′-azino-bis (3-ethylbenzothiazoline-6-sulfonic acid) or ABTS radical, cation decolorization activity/assay [14]. ABTS+, between 7 mM ABTS cation radical, was produced by the reaction in the water and 2.45 mM of the potassium persulfate (1:1) stored at room temperature in the dark for about 12–16 h before use. A dilution of ABTS solution with methanol was prepared to obtain 0.700 of absorbance at 734 nm. After adding 5 μL of the compound to the 3.995 mL diluted ABTS solution, 30 min after the starting and initial mixing, the absorbance was measured. In each assay, an appropriate solvent of blank was run. All the measurements were carried out in triplicate. Absorbance at about 734 nm percent inhibition was calculated by using the specific formula [14].

The formula for scavenging free radicals by a compound:

(1) $$\%\;Radical\;scavenging=\frac{A-B}{A}\times 100$$

A = control absorbance; B = sample abs.

Control value: A = 0.735, Abs. on UV–visible, spectrophotometer.

#### 2.5.3. Anti-Cholinesterase Assays

In these assays, the compound was analyzed spectrophotometrically for the inhibition potential of AChE and BuChE inhibition. As a substrate, the butyrylcholine iodide and acetylcholine iodide were used by following Ellman’s assay [15]. Dilutions of the compound were added in this assay to a cuvette that contained 5 μL of acetylcholinesterase in (0.03 µ/mL) and butyrylcholinesterase in (0.01 U/mL) in a 30 °C temperature water bath; about 5 μL of the DTNB catalyst was kept. Then, this was incubated for about 15 min. After the incubation phase, 5 μL of the substrate was added to the mixture to start the reaction. At 412 nm for about 4 min, absorbance was measured by using the double-beam spectrophotometer. 5-thio-2-nitrobenzoate anion was formed by a reaction between the DTNB and the thiocholines in yellow color. Feralolide was used as a control, while the reaction mixture had all of the above components. Percent enzymatic inhibition and percent enzymatic activity was calculated by a specific formula [15].

$$V=\frac{\Delta Abs}{\Delta t}$$

$$\%\;Enzyme\;activity=\frac{V}{Vmax}\times 100$$

% Enzyme inhibition=100−% Enzyme activity

(*V* (sample) shows the rate of reaction in the presence of an inhibitor and Vmax (control) shows the rate of reaction without inhibition.)

Control values: A1 = 0.734, A2 = 0.884, Abs. on UV–visible spectrophotometer.

### 2.6. Assessment of Behavioral Parameters in In Vivo Assays

#### 2.6.1. Assessment of Acute Toxicity Study of Feralolide

Feralolide was administered to five groups, each containing six animals. Group I served as a control, whereas groups II-V were given the compound at doses of 50, 100, 200, and 300 mg/kg, i.p. Behavioral properties of the feralolide were noted for the individual animal at 0, 30, 60, and 120 min, 24, 48, and 72 h, and one week after the administration of the drug. During the study, no acute toxicity signs were observed (evident from the absence of any effects on mortality, respiratory discomfort (cyanosis or gasping), altered reflex actions, as well as the lack of convulsions). A slight degree of sedation was observed at 200 and 300 mg/kg doses. Otherwise, all the animals seemed good 24 h to 1 week after the administration of the agents and no prominent alterations in the behavior, activities, and appearance were noted.

#### 2.6.2. Assessment of Antiamnesic Activity

##### Experimental Design

The treatment/administration of standard, drug, and test compounds in several groups was per protocol (Table 1). Randomly, the animals were put into six different groups, each group containing eight animals (*n* = 8). Each animal trial was marked with a permanent marker for easy identification. The volume of dose administrations was adjusted for all animals. For each behavioral experiment performed in this study, vehicle, feralolide, and the reference drug were administered to animals 1 h before each trial. The vehicle and the amnesic agent were administered 30 min before each trial [16].

#### 2.6.3. Novel Object Recognition Test (NORT)

According to the procedure of Bengoetxea and his coworkers, the novel object recognition task (NORT) was performed [17]. The apparatus was made of a white-colored box (40 cm, × 40 cm, ×66 cm) with a complex floor, which could be easily cleaned with 70% *v*/*v* of ethanol after each trial. A 60 W light was suspended in the apparatus, 50 cm over the wreck. NORT activity consisted of the habituation, sample, and test phases. In the sample phase, an individual mouse was positioned in an open field chamber with two identical objects (blue bottle) for about five minutes. Then, the mouse was returned to the home cage. The objects and the arena were cleaned with 70% *v*/*v* of ethanol in the middle of the trials to escape the olfactory cues. The test phase was conducted 24 h after the exposure of the sample phase. In the test phase, each mouse was again located in an open field chamber where one identical object had been exchanged with the novel object (red bottle). The object was placed in the arena so that 1/2 of the animals in each group looked at the novel object placed on the left side of the box arena, and the further half looked at the novel object on another or right side of the box arena, to remove preference of the sides. The time spent exploring each object in each phase was manually noted using a stopwatch. An animal was counted as exploring when its head was focused on the object within the expanse of about 2 cm or when the nose interacted with the specific object. Parameters assessed included the duration (in seconds) spent exploring the familiar object (TF), the time (in seconds) spent exploring the novel object (TN), and the total time (in seconds) spent exploring both objects (TF + TN). The percentage of the discrimination index (DI) was determined by using the following equation:

$$\;DI\left(\%\right)\frac{TN}{\left(TN+TF\right)}\times 100\%$$

#### 2.6.4. Assessment of Morris Water Maze Test (MWMT)

The Morris water maze test was used to test learning, including the acquisition of spatial memory, according to the method described previously [18,19]. By adding titanium dioxide, the water was made opaque. The tank was divided into four equal quadrants by using the two threads fixed at right angles on the rim of the pool to each other. At the center of the pool, the submerged platform was placed inside the tank, which was 1 cm below the water level. Throughout the training session, the position of the platform was unaltered. Each animal followed four consecutive trials each day with the different points within 5 min gaps between each of the trials for about four consecutive days, during which escape on the hidden platform was allowed and the animal was allowed to remain there for about 20 s. The animal was gently placed during the training session in the water from different locations and allowed 120 s to locate the submerged platform. If within 120 s the animal failed to locate the platform, it was guided on the platform gently and for 20 s the animal was allowed to remain there. On the retrieval day (last day) of the training session (i.e., Day 5), the platform was removed and the animal was placed into the pool from any point and allowed to explore the targeted quadrant for 300 s. In the center of the pool, the mean time spent searching for the missing platform by the rat in the center of the pool, which is an index of memory retrieval, was recorded.

#### 2.6.5. Assessment in Elevated Plus Maze (EPM)

Elevated plus maze is used extensively to evaluate cognition in rodents. The EPM arena or apparatus is based on the innate aversion of animals/rodents to open and high space. The procedure, technique, and end point for testing learning and memory were followed as per the parameters described earlier [20,21]. On the first day, each animal was placed at the end of an open arm, facing away from the central platform. Transfer latency (TL) was defined as the time required by the animal to move from the open arm into one of the closed arms with all four legs. TL was measured on the 1st day (i.e., 6th day of drug administration) for each animal (learning). If the animal did not enter into one of the closed arms within 90 s, it was gently pushed into one of the two closed arms and the animal was assigned the TL of 90 s. The animal was further allowed to explore the maze for another 2 min and then returned to its home cage. Retention of this learned task (memory) was evaluated after 24 h on the 7th day (trial day). A decline in the transfer latency of the animal serves as an indication of the memory-enhancing effect of the drug [22].

#### 2.6.6. Assessment in Passive Avoidance Test

In two equal-sized light and dark compartments, the passive avoidance test was carried out with the electrifiable floor grid in nature [23]. A guillotine door acted as the separation of the two compartments. In the acquisition trial phase, the mouse was initially placed in and allowed to explore the light compartment for about 40s. Upon opening the door into the dark compartment, the mouse moved and automatically the door was closed. The training trial was performed 24 h after the acquisition trial. Mice were allowed to explore the light compartment for about 40 s and then the guillotine door was opened. The door closed automatically as soon as the mice entered the dark compartment and, through the grid floor, an electric foot shock (0.1 mA/10 g body weight for 2 s) was delivered. All the drugs were administered after training to avoid modifying memory storage processes. A test trial was performed 24 h after the training trial using the same training program. The latency time was estimated by measuring the time before the mice entered the dark compartment after the opening of the door within the time of 180 s. If mice did not enter the dark compartment within 180 s, a latency time of 180 s was recorded.

#### 2.6.7. Video Recording

A camera installed in the animal house was used to record all the test proceedings and assess the experimental animals’ behavior.

### 2.7. Statistical Analysis

Graphpad prism^®^ was used for the statistical analysis in version 5 (Graph-Pad. Software Inc, San Diego, CA, USA). A Students–Newman–Keuls test for statistical significance calculation was followed by one-way ANOVA. Each value was expressed as the mean ± SEM and consideration of statistical significance was performed at *p* < 0.05.

### 2.8. Molecular Docking 

For molecular docking, the three-dimensional (3D) X-ray crystal structures of AChE (PDB: 1QTI, resolution = 2.50 Å) and BChE (PDB: 4B0O, resolution = 2.35 Å) were selected from the RCSB Protein Data Bank (RCSB PDB). Docking was performed in Molecular Operating Environment (MOE, version 2020.09). The 3D structure of the compound (feralolide) was drawn and optimized in MOE by minimizing its structure with an Amber12:EHT force field until the default parameters (RMS gradient = 0.1 Kcal/mol/Å). On the enzyme structures, hydrogen atoms were added and partial charges were calculated with an Amber12:EHT force field in MOE to prepare the files for docking. Previously, we studied the role of water molecules in the active site of AChE in detail [24]. Therefore, water molecules within 3 Å vicinity of the active site of AChE and BChE were retained in enzymes files, while the rest were removed. Moreover, heteroatoms other than co-crystallized ligands AChE and BuChE were also removed from protein files. Docking was carried out in MOE with its default parameters (triangle matcher docking algorithm and London dG scoring function).

## 3. Results

### 3.1. Chemistry

Feralolide was isolated as an amorphous powder that turned dark red on spraying with ceric sulfate. The molecular formula C_18_H_15_O_6_ was evidenced by LC-MS (ESI^+^), which exhibited molecular ion peaks at *m*/*z m*/*z* 345 [M + H]^+^. The ^1^H N.M.R. spectrum revealed the presence of two aromatic systems, each bearing a set of *m*-coupling protons (δ_H_ 6.29, 6.24, 6.20, 6.18, *J* = 2.2 Hz). One oxymethine multiplet (δ 4.72), protons of two methylene groups (δ_H_ 3.06/2.95 (*J* = 13.7, 7.4 Hz); δ_H_ 2.84/2.80 (*J* = 16.4, 10.4 Hz)) were confirmed by COSY to construct a 2-oxypropandiyl partial structure. The ^13^C-NMR/HMQC spectra delivered 18 carbon signals, two for ketone (δ_c_ 206.7) and ester carbonyls (δ_C_ 171.3). Based on HSQC/HMBC correlations, the oxymethine proton (δ_H_ 4.72) showed a cross signal with the carbon at C10 (δ_C_ 139.4) of the phenyl ring, confirming their linkage via CH_2_-9 (δ_C_ 39.6; δ_H_ 3.06, 2.95); hence, basic skeleton was confirmed. The phenyl ring was confirmed to bear an acetyl group at C-2′, while the residual two positions of C-3′ (δ 160.2) and C-5′ (δ_C_ 161.4) were established as phenolic carbons. The complete HMBC assignments confirmed the structure of the compound as a feralolide [25].

### 3.2. Assessment of Free Radical Scavenging Activities

#### 3.2.1. ABTS Free Radical Activity

In ABTS free radical scavenging assay, feralolide showed % ABTS inhibition of 70.96 ± 0.37, 67.56 ± 0.58, 49.72 ± 0.67, 42.12 ± 1.05, 28.99 ± 0.36, and 11.68 ± 0.56 with their IC50 value range of 210 μg/mL at a concentration of 1000, 500, 250, 125, 62.5, and 31.25 μg/mL. % ABTS inhibition of the feralolide was compared with the positive control, which was (ascorbic acid), and revealed a concentration, dependent reaction. Ascorbic acid indicated 83.94 ± 0.37 inhibitions at a concentration of 1000 μg/mL against the ABTS with the IC_50_ value range of 130 μg/mL (Table 2).

#### 3.2.2. DPPH Free Radical Activity

The DPPH free radical scavenging potential of the feralolide was 74.98 ± 0.29, 64.53 ± 0.18, 44.9 ± 0.25, 39.68 ± 0.19, 34.03 ± 0.19, and 29.87 ± 0.18 with the IC50 value range of about 270 μg/mL in the concentration range of about 1000, 500, 250, 125, 62.5, and 31.25 μg/mL (Table 3). The ascorbic acid indicated 86.78 ± 0.39 inhibitions at about 1000 μg/mL concentration against the DPPH with the IC_50_ value range of 63 μg/mL (Table 3).

#### 3.2.3. Inhibition of AChE Activity

Table 4 shows the results of the AChE inhibition by the various doses of the feralolide and the donepezil. Feralolide dose-dependently inhibited the AChE enzyme with the IC_50_ value range of 65 and 72 μg/mL, correspondingly. Likewise, the donepezil also inhibited the AChE enzyme with an IC50 value range of 60 and 67 μg/mL against the AChE, respectively (Table 4).

#### 3.2.4. Inhibition of BuChE Activity

Table 5 shows the results of the BuChE inhibition by the various doses of feralolide and donepezil. Feralolide dose-dependently inhibited the BuChE enzyme with the IC_50_ value range of 65 and 72 μg/mL, correspondingly. Likewise, the donepezil also inhibited the BuChE enzyme with an IC_50_ value range of 60 and 67 μg/mL against the BuChE, respectively (Table 5).

Percent enzymatic activity and percent enzymatic inhibition were calculated using the following formulas:

Formulas for compound:

$$V=\frac{\Delta Abs}{\Delta t}$$

$$\%\;Enzyme\;activity=\frac{V}{Vmax}\times 100$$

% Enzyme inhibition=100−% Enzyme activity

(*V* (sample) shows the rate of reaction in the presence of an inhibitor and *V_max_* (control) shows the rate of reaction without inhibition.)

Control values: A1 = 0.587, A2 = 0.745, Abs. on UV–visible spectrophotometer.

### 3.3. In Vivo Pharmacological Assessment

#### 3.3.1. Effects of Feralolide on the Acute Toxicity of Animals and Their General Behaviors

There was no significant toxic effect in acute toxicity testing, as was evident from the absence of respiratory discomfort (cyanosis or gasping), altered reflex actions, and the lack of convulsions. In four out of the six animals/mice, the escaping behavior and the spontaneous action were observed to be greater at doses of about 10 and 50 mg/kg. Additionally, there was an elevation in an allergic reaction (assessed as aggressive behavior during the treatment and showed a high increase in irritability) and the escape performance was seen as greater in the same animals. Five of the six animals/mice were observed to be slightly sleepy at 300 mg per kg. All the animals seemed good 24 h to 1 week after the administration/injection and no prominent alterations in the behavior, activities, and appearance were noted.

#### 3.3.2. Effect of Feralolide in Novel Object Recognition Test

**Figure 2:**
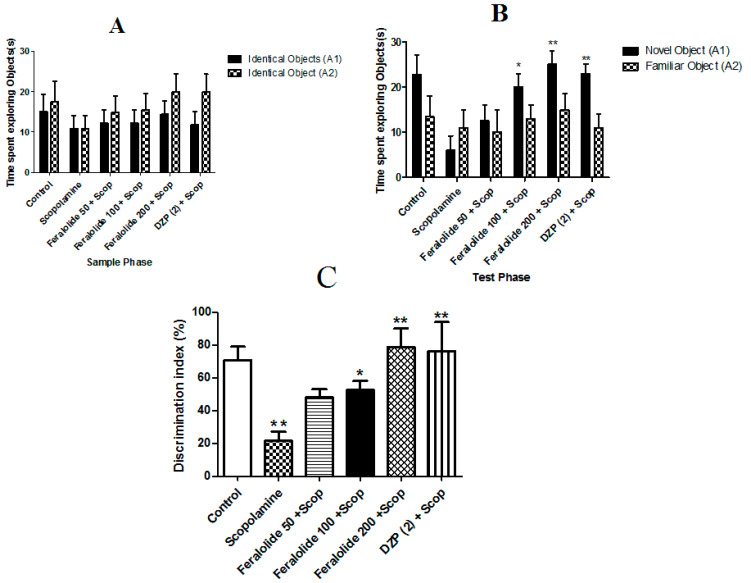
Effect of feralolide (50, 100, and 200 mg/kg) in a short-term memory loss NORT. (**A**) exploration duration in sample phase, (**B**) exploration duration in test phase, (**C**) the discrimination index (DI). * *p* < 0.01. vs. the control group and * *p*, < 0.05, ** *p*. < 0.01 vs. the scopolamine 1 mg/kg.

The results obtained with the novel object recognition test are shown in Figure 2. In the sample phase, no significant difference was observed in the total time spent exploring both objects (Figure 2A). Furthermore, no significant difference was found between feralolide and scopolamine-treated groups in exploring each identical object.

However, in the test phase, the group treated with the feralolide at the doses of 100 and 200 mg/kg + scopolamine (1 mg/kg) and donepezil (2 mg/kg) and scopolamine (1 mg/kg) spent a significantly longer time with the novel object than the familiar one compared with the scopolamine-treated group only (* *p* < 0.05; ** *p* < 0 0.01 respectively) (Figure 2B).

A significant dose-dependent increase was noted in the percentage discrimination index (% DI) with feralolide at the doses of 100, 150, and 200 mg/kg. There was also a significant dose-dependent increase in the percentage discrimination index (% DI) with feralolide at 100 and 200 mg/kg (*p* < 0.05; *p* < 0 0.01, respectively) compared with scopolamine. Donepezil (2 mg/kg) also caused a significant increase in the % DI (*p* < 0.01) (Figure 2C).

#### 3.3.3. Effect of Feralolide in Morris Water Maze Test (MWMT)

**Figure 3:**
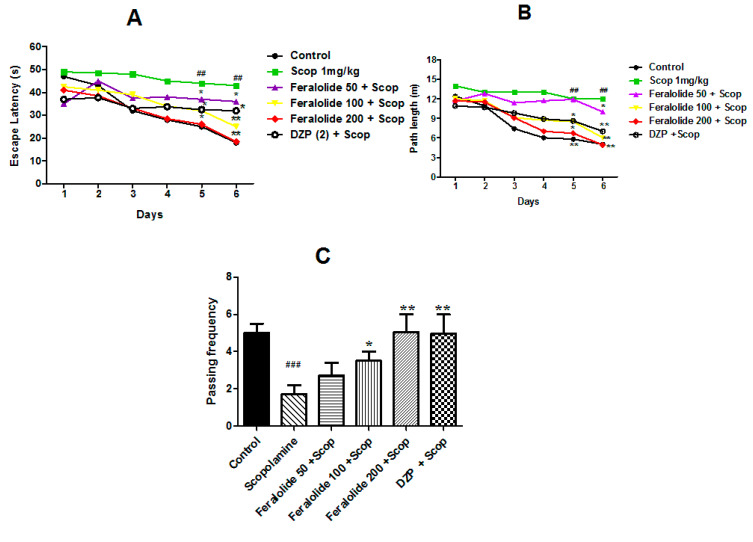
Effects of the feralolide (50, 100, and 200 mg/kg) on scopolamine-induced cognitive/memory impairment in mice. Memory impairment was carried out by the administration of scopolamine at the dose of (1 mg/kg, i.p). (**A**) Mice escape latency in the hidden platform (tests for 6 consecutive days); (**B**) mice path length in the hidden platform tests (6 consecutive days); (**C**) mice frequency passing through hidden platform location. * *p* < 0.05; ** *p* < 0.01; against the scopolamine administered group. ## *p* < 0.01, ### *p* < 0.05; against the control group.

Morris’s water maze test is profound in assessing dysfunction in spatial memory and learning. All groups’ mice performance was improved through the training phase, as shown by the reduced escape latency over the consecutive days (Figure 3A). A significant modification was found in mean latency between the training days (F (4, 116) = 34.32, *p* < 0.001) and between treatments (F (5, 119) = 26.31, *p* < 0.001). Still, there was no interaction observed between the training day and the groups (F (20, 016) = 0.738, *p* > 0.05), proposing that alterations among the different groups were dependent on treatment. The hidden platform–swimming trials showed their escape latency, and their path length in the scopolamine-induced group animal/mice was significantly increased when compared with the control group on the fifth and sixth testing days (*p* < 0.01, *n* = 8; Figure 3A,B). In contrast, treatment with the feralolide significantly decreased the escape latency and path length at the doses of 50, 100, and 200 mg/kg on the fifth and sixth day (*p* < 0.05, *p* < 0.01, *n* = 8) when compared with the scopolamine treated group. Similar effects were observed with donepezil at 2 mg/kg. On the sixth day after the last training trial, the mice were subjected to the probe test, in which the platform was removed from the pool to conclude whether the mice could recall the platform’s location. In comparison with the control group animal/mice, the spatial memory of the scopolamine-induced mice was significantly damaged, passing through the less target zone where the hidden platform was located, compared with the control group (Figure 3C, ### *p* < 0.05). In contrast, the feralolide and donepezil-treated groups spent significantly less time in the target quadrant than the scopolamine-treated amnesic animals (* *p* < 0.05, ** *p* < 0.01, *** *p* < 0.001, *n* = 8).

#### 3.3.4. Assessment of Feralolide in Elevated Plus Maze Test (EPM)

**Figure 4:**
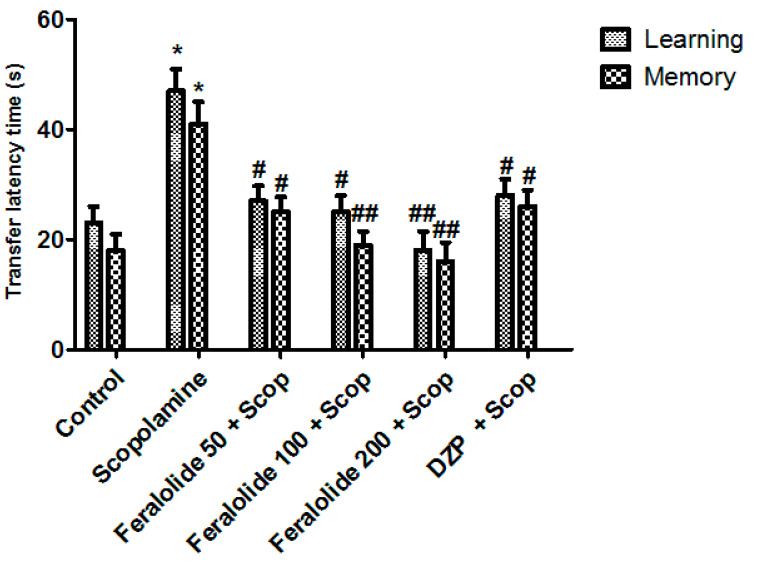
Effect of feralolide on learning and memory in EPM. Donepezil (2 mg/kg) was used as a standard drug. * *p* < 0.01 compared with control animals, # *p* < 0.0, ## *p* < 0.01 compared with scopolamine-treated animals. Values are mean ± SEM (*n* = 8), ANOVA followed by Tukey–Kramer test.

The transfer latency (TL) on a second day reveals the retaining of the learned task or the memory. Scopolamine (1 mg/kg, i.p.) given before the training significantly increased (*p* < 0.01) the transfer latency (TL) on the first and second day, showing impairment in both learning and cognition or memory compared with the control (* *p* < 0.05). All animals treated with the feralolide in doses of (50, 100, and 200 mg/kg; i.p.) revealed a dose-dependent decrease in TL on the sixth day compared with the scopolamine group (learning) (# *p* < 0.05, ## *p* < 0.01) (Figure 4). Donepezil also caused a significant decrease in the TL compared with the scopolamine-treated group. Feralolide and donepezil also significantly decreased the TL time on the seventh day (memory) of drug administration compared with the scopolamine-treated group, indicating a memory-enhancing effect (# *p* < 0.05, ## *p* < 0.01).

On the first day, TL was noted (training session) for each animal. The animal was permitted to discover the elevated plus maze for another two minutes and then reverted to its home cage. Twenty-four hours later (trial day), the animals were tested again for transfer latency. The transfer latency was measured when the mouse or animal moved from the open arms into the closed arms with all four legs. A substantial decrease in the TL indicated an improvement in cognition or memory.

#### 3.3.5. Effect of Feralolide on Passive Avoidance Test (PAT)

**Figure 5:**
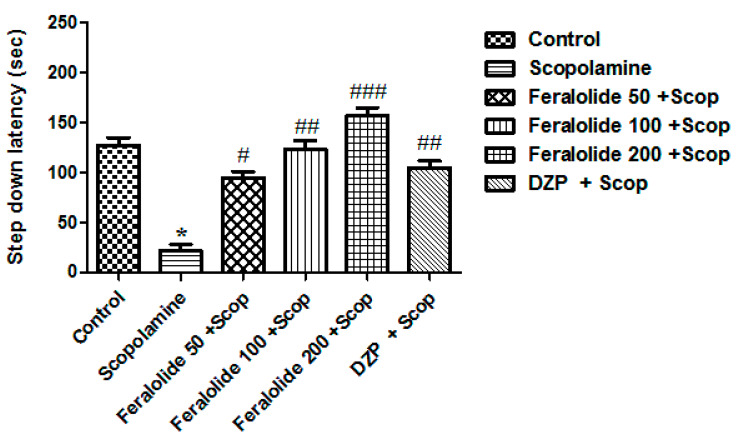
Effect of feralolide on the SDL in passive avoidance test. * *p* < 0.05 compared with scopolamine, # *p* < 0.05, ## *p* < 0.01, ### *p* < 0.001 compared with scopolamine. Value ranges are mean ± SEM (*n* = 8), ANOVA; followed by the Tukey–Kramer test. Donepezil (2 mg/kg) was used as a standard reference drug.

Step-down latency (SDL) on the second day echoed the long-term memory of the animals/mice. Various doses of the feralolide (50, 100, and 200 mg/kg) administered to the mice showed a dose-dependent elevation in the SDL value range; they significantly overturned the memory deficits due to the amnesia induced by scopolamine (*p* < 0.05, *p* < 0.01, *p* < 0.001) compared with the scopolamine-treated group. (Figure 5). Animals treated with donepezil (2 mg/kg) also showed significant enhancement in scopolamine-induced memory deficits (## *p* < 0.01).

#### 3.3.6. Molecular Docking of Feralolide in AChE and BuChE

**Figure 6:**
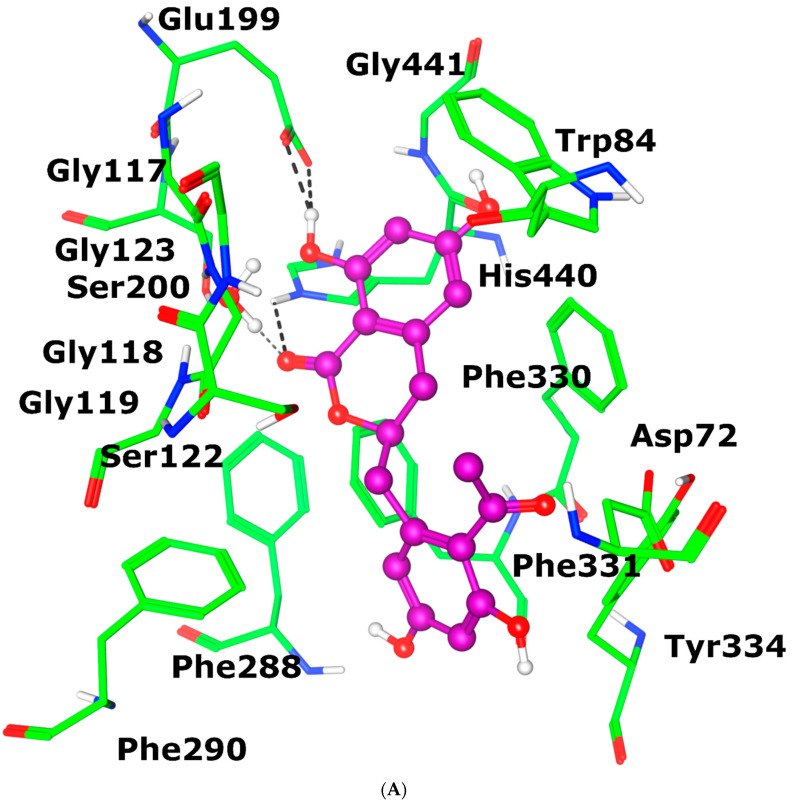

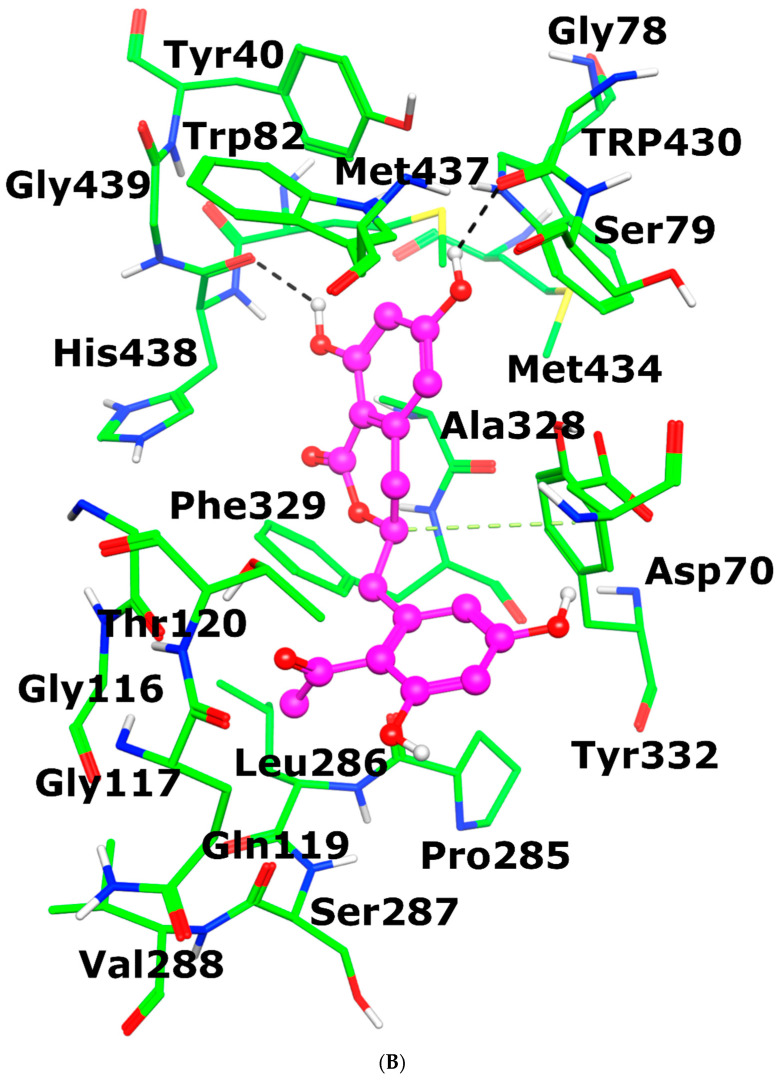
The compound is shown in the magenta ball and stick model; active site residues of enzymes are depicted in the green stick model, H-bonds are shown in black dotted lines, and hydrophobic interaction is displayed in light green dotted line. The binding mode of the compound is shown in the active sites of AChE (**A**) and BuChE (**B**).

The flavone moiety of the compound is responsible for binding interaction with the active site residues of AChE. The compound’s carbonyl and hydroxyl functional groups mediate strong H-bonds with the side chains of His440 (2.47 Å) and Glu199 (2.40 Å and 2.01 Å), respectively. In contrast, one water molecule in the vicinity (WAT820) also interacted with the carbonyl oxygen of the flavone ring at 1.66 Å. Due to these interactions, the compound produced a highly negative docking score, i.e., −7.24 kcal/mol. The binding mode of the compound in the active site of AChE is shown in Figure 6A.

When feralolide was docked in the active site cavity of BuChE, the hydroxyl groups at the flavone ring of the compound showed H-bonds with the main chain carbonyl groups of Gly78 and His438 at 2.0 Å and 2.26 Å, respectively, while the flavone ring of the compound also mediated a hydrophobic interaction (π-H) with the side chain of Tyr332. The compound exhibited a slightly higher docking score in the active site of BuChE, i.e., −7.82 kcal/mol, than in the binding cavity of AChE. The docking scores correlated well with the IC_50_ values of the compound. The docked view of feralolide in the active site of BuChE is shown in Figure 6B.

## 4. Discussion

Several authors have described aloe species as having an inhibitory action on the acetylcholinesterase (AChE) and butyrylcholinesterase (BuChE) [26,27] that results in the elevation of long-term recognition or memory. Thus, the agents having an inhibitory effect on these enzymes may be beneficial for the treatment of memory dysfunctions, including Alzheimer’s disease. In the treatment of Alzheimer’s disorder and other dementia disorders, choline esterase reversible inhibitors have been used as a cognitive enhancer.

Flavonoids have been shown to cross the blood–brain barrier, stabilizing brain activities and withdrawing the memory and cognitive weakness effect of scopolamine [28]. In this research study, feralolide, a flavone isolated from the *Aloe ferox* miller, inhibited the AChE and BuChE with the IC_50_ of 55 and 52 μg/mL, respectively. The IC_50_ value ranges of the feralolide and donepezil were not significantly different against the acetylcholinesterase (AChE) and the butyrylcholinesterase (BuChE). In age-related cognitive and memory deficits, oxygen free radicals are involved, and they may be accountable for memory deterioration in Alzheimer’s disease that occurs in old age people [29]. Antioxidants are known to counteract the attack of free radicals and may be beneficial to increase cognitive or memory functions in Alzheimer’s disease. Currently, research has been focussed on the use of antioxidants from natural sources, particularly plants, to protect the human body, especially the oxidative damage of the brain tissues caused by free radicals. Feralolide, as apparent from the findings of the current study, indicates antioxidant potential in the DPPH and the ABTS free radical scavenging assay.

In experimental animals and humans, scopolamine causes memory impairment, causing impairment in giving out information, keeping consideration, and gaining novel knowledge [30]. Thus, in test subjects, scopolamine-prompted amnesia is a generally referred model for the simulation of human dementia as an imperative and investigational Alzheimer’s disease [31].

Scopolamine-induced memory impairment is most commonly used to assess the antiamnesic effect potential of therapeutic substances in experimental animals. For example, ginkgo ketoester tablets (GT) and donepezil—a clinically used combination for the treatment of AD—have been evaluated and validated using the scopolamine-induced memory impairment model [32]. Similarly, other agents have also been found to possess significant improvements in memory impairment in this model [33]. Scopolamine induces learning and memory deficits by blocking cholinergic signaling [34].

In the present study, the Morris water maze, elevated plus maze, passive avoidance, and novel object recognition tests were carried out to identify the memory-improving effects of the feralolide in the scopolamine-induced memory impairment in mice. The escape latency of repeated trial tests for six days and the time spent in the target quadrant in the probe test were investigated in the Morris water maze test. The results obtained in the MWM tests indicated that feralolide significantly attenuated scopolamine-induced memory impairment and improved long-term spatial memory in the MWM test. Similarly, in EPM, feralolide reversed scopolamine-induced decreases in transfer latency, thus suggesting improvement in learning and memory. The passive avoidance test is based on the principle that the animal learns to avoid an aversive stimulus such as an electrical foot shock. Administration of feralolide ameliorated the scopolamine-induced memory deficit by elevating the SDL in the passive avoidance test, suggesting that it may improve long-term memory. The results obtained in the NORT were consistent with the findings of the MWM, EPM, and passive avoidance tests. Feralolide caused a dose-dependent increase in the exploration time of the novel object in the test phase and discrimination index, whereas scopolamine failed the novelty test, indicating that feralolide prevents the memory impairment induced by scopolamine.

The memory-enhancing effects of feralolide observed in this study may be due to the inhibition of the AChE and BuChE enzymes involved in the degradation of ACh. Thus, agents inhibiting these enzymes can cause an elevation in the level of ACh. Furthermore, the effects observed in this study were similar to donepezil, a standard AChE inhibitor, suggesting that the mechanism (s) of action involved in the effect of feralolide may be identical to the mechanisms of donepezil [35].

## 5. Conclusions

In conclusion, feralolide exerts antioxidant effects, inhibits choline esterase enzymes, and exhibits significant antiamnesic effects in a battery of in vivo behavioral paradigms. Thus, feralolide could be a useful novel agent for the development of therapeutic alternatives for treating memory dysfunction in Alzheimer’s disease.

## Figures and Tables

**Table 1:** Various treatment groups were used in the study.

Group	Group Category	Test Solution	Route
Group I	Normal control	Vehicle 8 mL/kg	p.o.
Group II	Negative control	Scopolamine 1 mg/kg	i.p.
Group III	Positive control	Donezepil 2 mg/kg + scopolamine 1 mg/kg	p.o/i.p
Group IV	Treatment group-1	Feralolide 1 mg/kg + scopolamine 1 mg/kg	p.o/i.p
Group V	Treatment group-2	Feralolide 5 mg/kg + scopolamine 1 mg/kg	p.o/i.p
Group VI	Treatment group-3	Feralolide 10 mg/kg + scopolamine 1 mg/kg	p.o/i.p

**Table 2:** ABTS assay activity results. Values represent means ± standard error of mean of triplicate readings (*n* = 3).

Sample	Concentration (μg/mL)	% ABTS Scavenging Mean ± SEM	IC_50_ (µg/mL)
Feralolide	1000 µg/mL	70.96 ± 0.37	**170**
	500 µg/mL	67.56 ± 0.58	
	250 µg/mL	49.72 ± 0.67	
	125 µg/mL	42.12 ± 1.05	
	62.5 µg/mL	28.99 ± 0.36	
	31.25 µg/mL	11.68 ± 0.56	
Ascorbic acid	1000 µg/mL	83.94 ± 0.47	**130**
	500 µg/mL	74.68 ± 0.28	
	250 µg/mL	63.98 ± 1.00	
	125 µg/mL	49.67 ± 0.57	
	62.5 µg/mL	45.79 ± 1.09	
	31.25 µg/mL	39.73 ± 0.65	

**Table 3:** DPPH assay activity results. Values represent means ± standard error of mean of triplicate readings (*n* = 3).

Sample	Concentration (μg/mL)	% DPPH Scavenging Mean ± SEM	IC50 (µg/mL)
Feralolide	1000 µg/mL	74.98 ± 0.29	220
	500 µg/mL	64.53 ± 0.18	
	250 µg/mL	44.9 ± 0.25	
	125 µg/mL	39.68 ± 0.19	
	62.5 µg/mL	34.03 ± 0.19	
	31.25 µg/mL	29.87 ± 0.18	
Ascorbic acid	1000 µg/mL	86.78 ± 0.40	63
	500 µg/mL	74.9 ± 0.35	
	250 µg/mL	67.98 ± 0.37	
	125 µg/mL	52.87 ± 0.86	
	62.5 µg/mL	46.95 ± 0.18	
	31.25 µg/mL	30.29 ± 1.10	

**Table 4:** AChE assay activity results. Values represent means ± standard error of mean of triplicate readings (*n* = 3).

Sample	Concentration (μg/mL)	% AChEI Scavenging Mean ± SEM	IC50 µg/mL
Feralolide	1000 µg/mL	86.91 ± 0.43	55
	500 µg/mL	84.21 ± 0.32	
	250 µg/mL	76.98 ± 0.35	
	125 µg/mL	61.89 ± 0.66	
	62.5 µg/mL	48.88 ± 0.54	
	31.25 µg/mL	30.73 ± 0.33	
Donepezil	1000 µg/mL	96.08 ± 0.43	60
	500 µg/mL	94.03 ± 0.32	
	250 µg/mL	84.08 ± 0.23	
	125 µg/mL	72.09 ± 0.16	
	62.5 µg/mL	49.06 ± 0.15	
	31.25 µg/mL	19.50 ± 0.23	

**Table 5:** BuChE assay activity results. Values represent means ± standard error of mean of triplicate readings (*n* = 3).

Sample	Concentration (μg/mL)	% BuChEI Scavenging Mean ± SEM	IC50 µg/mL
Feralolide	1000 µg/mL	79.8 ± 0.39	52
	500 µg/mL	68.9 ± 0.30	
	250 µg/mL	61.73 ± 0.39	
	125 µg/mL	52.68 ± 0.69	
	62.5 µg/mL	37.98 ± 0.38	
	31.25 µg/mL	24.93 ± 0.29	
Donepezil	1000 µg/mL	93.39 ± 0.48	67
	500 µg/mL	88.95 ± 0.30	
	250 µg/mL	79.9 ± 0.29	
	125 µg/mL	68.9 ± 0.58	
	62.5 µg/mL	41.34 ± 0.48	
	31.25 µg/mL	24.93 ± 0.32	

## Data Availability

The data are contained within the article.
